# Identification, Cloning, and Functional Characterization of Carotenoid Cleavage Dioxygenase (CCD) from *Olea europaea* and *Ipomoea nil*

**DOI:** 10.3390/biology14070752

**Published:** 2025-06-24

**Authors:** Kaixuan Ke, Yufeng Zhang, Xinyi Wang, Zhaoyan Luo, Yangyang Chen, Xianying Fang, Linguo Zhao

**Affiliations:** 1 National Key Laboratory for the Development and Utilization of Forest Food Resources, Nanjing Forestry University, Nanjing 210037, China; kainj98c@gmail.com (K.K.); zhangyf0430@163.com (Y.Z.); wxy5787@163.com (X.W.); 15693377009@njfu.edu.cn (Z.L.); 13862757724@163.com (Y.C.); 2 College of Chemical Engineering, Nanjing Forestry University, Nanjing 210037, China; 3 Jiangsu Co-Innovation Center of Efficient Processing and Utilization of Forest Resources, Nanjing Forestry University, Nanjing 210037, China; 4 Jinpu Research Institute, Nanjing Forestry University, Nanjing 210037, China

**Keywords:** carotenoid cleavage dioxygenases, CCD1 family, enzyme characterization, β-ionone

## Abstract

The aromatic C_13_ apocarotenoid β-ionone is a high-value natural-flavor and -fragrance compound derived from the oxidative cleavage of carotenoids. Carotenoid cleavage dioxygenases (CCDs) play a pivotal role in the biosynthesis of volatile apocarotenoids, particularly β-ionone. In this study, we report the identification, cloning, and functional characterization of two CCD1 homologs: *OeCCD1* from *Olea europaea* and *InCCD1* from *Ipomoea nil*. These two species, which, respectively, represent a woody perennial and a herbaceous annual, were selected to explore the potential functional divergence of CCD1 enzymes across different plant growth forms. These CCD1 genes were synthesized using codon optimization for *Escherichia coli* expression, followed by heterologous expression and purification using a GST-fusion system. In vitro assays confirmed that both enzymes cleave β-carotene at the 9,10 (9′,10′) double bond to yield β-ionone, but only *OeCCD1* exhibits detectable activity on zeaxanthin; *InCCD1* shows no in vitro cleavage of zeaxanthin. Kinetic characterization using β-apo-8′-carotenal as substrate revealed, for *OeCCD1*, a K_m_ of 0.82 mM, V_max_ of 2.30 U/mg (k_cat_ = 3.35 s^−1^), and k_cat_/K_m_ of 4.09 mM^−1^·s^−1^, whereas *InCCD1* displayed K_m_ = 0.69 mM, V_max_ = 1.22 U/mg (k_cat_ = 1.82 s^−1^), and k_cat_/K_m_ = 2.64 mM^−1^·s^−1^. The optimization of expression parameters, as well as the systematic evaluation of temperature, pH, solvent, and metal ion effects, provided further insights into the stability and functional diversity within the plant CCD1 family. Overall, these findings offer promising enzymatic tools for the sustainable production of β-ionone and related apocarotenoids in engineered microbial cell factories.

## Simple Summary

β-Ionone is a valuable aroma compound widely used in the fragrance, food, and pharmaceutical industries. In this study, we identify and characterize two carotenoid cleavage dioxygenase 1 (CCD1) genes from *Olea europaea* and *Ipomoea nil*. By expressing these genes in *E. coli*, we evaluate their ability to produce β-ionone and analyze their biochemical properties. Our findings not only provide insights into the functional diversity of CCD1 enzymes across plant species, but also offer new genetic resources for the microbial biosynthesis of natural aroma compounds.

## 1. Introduction

Carotenoids serve multifaceted biological roles in nature, functioning not only as essential regulatory phytohormones, but also as precursors for volatile aromatic compounds and vivid chromophores [1,2,3,4]. Among plant-derived volatile carotenoid derivatives, β-ionone has garnered particular attention due to its distinctive properties and applications [5]. Characterized by a 13-carbon monocyclic terpenoid skeleton, β-ionone is ubiquitously distributed across various plant taxa, notably *Osmanthus fragrans*, *Vitis vinifera*, and *Camellia sinensis* [6]. This compound exhibits an exceptionally low olfactory detection threshold in both aqueous and gaseous phases, presenting characteristic sensory notes of warm woodiness, berry undertones, and violet florals. Regulatory agencies, including China’s National Health Commission (2014), the U.S. Flavor and Extract Manufacturers Association (FEMA), and the Food and Drug Administration (FDA), have approved β-ionone as a safe synthetic flavoring agent [7]. Beyond alimentary applications, it has extensive utilization in cosmetic formulations (e.g., premium perfumes, shampoos), personal care products, and household cleaning agents. Pharmacological investigations further reveal its broad spectrum of bioactivities, encompassing anticancer, anti-inflammatory, antiviral, antioxidant, antimicrobial, antihelmintic, and hypolipidemic properties [8].

Although the chemical synthesis of β-ionone remains technically feasible, this approach faces increasing challenges from market demands for natural-sourced alternatives, thereby increasing the demand and commercial value of biologically derived β-ionone [6,9]. Conventional synthesis routes involving pseudoionone cyclization require stringent reaction conditions with strong acids, often yielding undesirable byproducts and demonstrating poor selectivity. Subsequent isomerization and oxidation steps necessitate meticulous process control to ensure product purity [6,9]. Extraction methodologies, including steam distillation and solvent-based techniques, present inherent limitations, such as energy intensiveness, thermal degradation risks, and reliance on toxic solvents, requiring complex purification [9]. In light of these challenges, contemporary research has identified alternative biosynthetic pathways by using the oxidative transformation of C40 carotenoids [10,11,12,13,14], catalyzing a heightened interest in biotechnological production strategies that leverage enzymatic bioconversion and synthetic biology platforms.

The enzymatic cleavage of carotenoids, mediated by carotenoid cleavage dioxygenases (CCDs), represents an evolutionarily conserved biochemical mechanism spanning the bacterial, fungal, plant, and animal kingdoms [12]. Nevertheless, efficient β-ionone biosynthesis confronts significant bottlenecks, particularly the catalytic constraints imposed by CCDs [5,11,14,15]. Phylogenetic analyses categorize CCDs into seven distinct subfamilies: CCD1, CCD2, CCD4, CCD7, CCD8, CCD10, and 9-cis-epoxycarotenoid dioxygenase (NCED) [16,17,18,19,20,21]. Subcellular localization studies reveal the cytoplasmic distribution of CCD1 versus plastid-localized CCD2/4/7/8/NCED, with CCD10 exhibiting dual compartmentalization [22,23,24]. Functional diversification among these isoforms introduces complexity to β-ionone biosynthesis. Current research emphasizes two predominant plant CCD subfamilies (CCD1 and CCD4) capable of catalyzing C9-C10/C10′-C9′ double-bond cleavage in β-carotene to generate β-ionone [17,25,26]. Notably, comparative enzymatic analyses demonstrate substantial variation in substrate specificity and catalytic efficiency across CCD variants. In a comprehensive screening of twelve CCD enzymes (nine CCD4 and three CCD1 isoforms) expressed in *E. coli* ε-carotene systems, CCD4 homologs generally exhibited inferior activity relative to CCD1 counterparts, underscoring functional heterogeneity within the CCD superfamily [15]. Since the seminal identification of *Arabidopsis thaliana* CCD1 (*AtCCD1*) in 2001 [27], subsequent studies have achieved significant progress in CCD1 engineering and characterization from diverse sources [14]. However, substantial inter-species variability in catalytic performance persists among CCD1 orthologs, necessitating the systematic exploration of novel enzymes.

Given the observed functional variability among CCD1 orthologs, we hypothesized that evolutionary differences between woody and herbaceous plants may contribute to distinct catalytic characteristics. To test this, we selected and characterized CCD1 genes from *Olea europaea* (a perennial woody plant) and *Ipomoea nil* (a fast-growing herbaceous species), aiming to compare their enzymatic profiles in the context of β-ionone biosynthesis.

To address these limitations, this investigation systematically evaluates two CCD1 homologs—*OeCCD1* (from *Olea europaea*) and *InCCD1* (from *Ipomoea nil*)—using a multidisciplinary approach. Experimental workflows encompassed the following: (1) gene identification and molecular cloning; (2) heterologous expression in *E. coli*; (3) in vitro/vivo functional validation; (4) expression kinetics and biochemical profiling; and (5) the evaluation of the potential for high-yield β-ionone biosynthesis. Our findings establish these CCD1s as versatile biocatalysts, providing fundamental insights for CCD resource expansion, structure–function elucidation, and synthetic biology applications. This work lays critical groundwork for the engineering of CCD variants and the development of cell factory platforms that are aimed at the high-efficiency production of volatile aroma compounds from carotenoid precursors.

## 2. Experimental Section

### 2.1. Chemicals

β-Ionone, β-apo-8′-carotenal, lycopene, β-carotene, zeaxanthin, lutein, astaxanthin, and all-trans-retinal were acquired from Aladdin Biochemical Technology Co., Ltd. (Shanghai, China). Molecular biology reagents, such as isopropyl-β-D-thiogalactoside (IPTG), chloramphenicol, and ampicillin, were sourced from Sangon Biotech (Shanghai, China). High-performance liquid chromatography (HPLC)-grade solvents, specifically ethyl acetate, methanol, and acetone, were procured from Sigma-Aldrich Co., Ltd. (Shanghai, China). All other reagents used were of analytical grade and were obtained from reputable commercial sources.

### 2.2. Plasmid Construction and Sequence Analysis of CCD1s

All strains and plasmids used in this study are listed in Table S1. The carotenoid cleavage dioxygenase gene *OeCCD1* (GenBank accession number XM_023040081.1) from *Olea europaea* and *InCCD1* (GenBank accession number XM_019295309.1) from *Ipomoea nil* underwent synthetic engineering, incorporating codon optimization tailored for expression in *E. coli*. To facilitate molecular cloning, recognition sites for the restriction enzymes *Bam*H I and *Eco*R I were strategically introduced at the 5′ and 3′ termini of the CCD1 coding sequence, respectively. Following enzymatic digestion with *Bam*H I and *Eco*R I, the CCD1 genes were ligated into the bacterial expression vectors pETDuet-1 and pGEX-2T-1, generating the recombinant constructs pETDuet-1-CCD1s and pGEX-2T-CCD1s, respectively.

Phylogenetic relationships were elucidated through the construction of a neighbor-joining (NJ) tree using MEGA 11 software. Multiple sequence alignments were conducted using Clustal W, enabling a comparative genomic analysis of CCD1s with homologous sequences.

### 2.3. Protein Expression and Purification

The recombinant plasmid pGEX-2T-CCD1s were successfully transformed into *E. coli* BL21 (DE3) using the heat shock method. For protein expression induction, the transformed *E. coli* cells were initially cultured under stationary conditions at 37 °C overnight, then inoculated into 50 mL of Luria–Bertani (LB) broth and incubated at 37 °C with continuous agitation at 180 rpm. Upon reaching an optical density (OD600) within the range of 0.6–0.8, expression was induced by adding IPTG to a final concentration of 0.1 mM, followed by further incubation at a reduced temperature of 28 °C for 21 h to optimize protein yield.

In the subsequent phase, culture conditions for *E. coli* BL21 (DE3) harboring pGEX-2T-CCD1s were refined to maximize expression efficiency. Post-cultivation, cells were collected by centrifugation at 10,000× *g* for 20 min at 4 °C, resuspended in 5 mL of 1× Phosphate-Buffered Saline (PBS, pH 7.4) containing 5 mM Na-ascorbate to prevent oxidative damage, and subjected to cell lysis via ultrasonication under ice-cold conditions to minimize thermal denaturation. Cellular debris was then removed by additional centrifugation at 10,000× *g* for 40 min at 4 °C.

Purification of the GST-tagged recombinant protein was achieved through GST affinity chromatography, following the protocols provided by Sangon Biotech (Shanghai, China) [28]. The integrity of the purified protein was assessed via Sodium Dodecyl Sulfate–Polyacrylamide Gel Electrophoresis (SDS-PAGE). Protein concentration was quantified using the Bradford assay, with Bovine Serum Albumin (BSA) as a calibration standard, according to the guidelines provided by Sangon Biotech (Shanghai, China).

### 2.4. Preparation of Carotenoids for In Vitro Assays

The methodology for carotenoid preparation was adapted from a protocol described in the prior literature, with minor modifications [29]. Carotenoid substrates were adjusted to a final concentration of 40 μM for experimental procedures. To facilitate carotenoid micelle formation, substrates were first dissolved in 50 μL of dichloromethane, followed by the addition of 50 μL of Triton X-100 in ethanol solution, maintaining a 1:100 molar ratio relative to the carotenoid substrates. This homogenized mixture was then subjected to vacuum centrifugation for desiccation. The desiccated residues were reconstituted in ultrapure water and stored at 4 °C until they were required for subsequent experiments.

### 2.5. Substrate Specificity Assays In Vitro

Experiments to elucidate the substrate specificity of CCD1s were conducted in accordance with a methodology previously described in the literature, with slight modifications for adaptation to the current study’s objectives. Reaction volumes were standardized to 1 mL, comprising an optimized sodium phosphate buffer, the designated substrate, and 100 μL of the CCD1 enzyme. This reaction mixture was then incubated at a constant temperature of 35 °C for an overnight duration. Subsequent to the incubation period, 0.5 mL of chloroform was introduced to the reaction milieu, functioning dually as an enzymatic inhibitor and as a solvent for the extraction of assay products.

For the analytical determination of C13 compounds, a Trace ISQ-LT Gas Chromatography–Mass Spectrometry (GC-MS) system (Thermo Fisher Scientific, Waltham, MA, USA) was utilized. The system was equipped with a DB-5MS analytical column (dimensions: 30 m × 0.25 mm × 0.25 μm) and integrated with a flame ionization detector. The operational interface temperature was meticulously maintained at 250 °C. Electron impact ionization (EI-MS) methodology was employed, utilizing an ionization energy of 70 eV, and the mass spectrometric analysis was conducted over a range spanning from 50 to 450 *m*/*z*. Spectral data acquisition and subsequent analyses were performed using the Xcalibur software, version 1.4, facilitating a comprehensive evaluation of the generated mass spectra.

### 2.6. Two-Plasmid System for In Vivo Tests on Carotenoid Cleavage Activity

*E. coli* strains, molecularly engineered to facilitate the biosynthesis of specific carotenoids, namely β-carotene (incorporating plasmid pAC-BETA, Addgene plasmid #53272) and zeaxanthin (utilizing plasmid pAC-ZEAX, Addgene plasmid #53274), were subjected to transformation with either the target expression vector pETDuet-1-CCD1s or the comparative control vector pETDuet-1.

Recombinant protein synthesis in these genetically modified organisms was induced by administering 0.1 mM IPTG. Volatile organic compounds were subsequently collected using Solid Phase Microextraction (SPME) and analytically characterized via GC-MS, following the protocols described in the existing scientific literature.

The extraction of carotenoids from either the bacterial cellular matrices or the cultivation medium was executed according to previously validated methodologies. Analytical quantification and qualitative assessment of the carotenoids were conducted using HPLC (1200 Infinity II, Agilent Technologies, Little Falls, DE, USA). This analysis employed a reversed-phase Zorbax Eclipse Plus C18 column (5 μm particle size, dimensions 1.5 × 4.6 mm, Agilent Technologies, Little Falls, DE, USA). Chromatographic separation was facilitated using a mobile-phase composition of methanol, ethyl acetate, and water in a volumetric ratio of 50%/48%/2% (*v*/*v*/*v*), under isocratic conditions, at a flow rate of 1 mL/min over a period of 20 min at a controlled temperature of 30 °C. The detection of carotenoids was achieved at a Diode Array Detector (DAD) wavelength of 450 nm, enabling the precise identification and quantification of these phytochemicals.

### 2.7. Enzymatic Activity Assay of Recombinant CCD1

The enzymatic activity of recombinant CCD1 was evaluated using β-apo-8′-carotenal as the substrate. The standard reaction system consisted of 100 mM phosphate buffer (pH 7.0), β-apo-8′-carotenal (40 μM), 200 μL of pure enzyme, and water, and was incubated at 30 °C in a water bath for 20 min.

The enzymatic activity of recombinant CCD1 (U) is defined as the amount of enzyme required to catalyze the formation of 1 nmol of β-ionone from β-apocarotenoid-8′-aldehyde per minute. Enzymatic activity was calculated with reference to a standard curve of β-ionone.

### 2.8. Study of the Enzyme Properties of Recombinant CCD1

#### 2.8.1. Determination of Optimum Temperature and Temperature Stability

A temperature gradient ranging from 30 °C to 65 °C was established to assess the catalytic activity of the enzyme under different temperatures. The highest measured enzyme activity was defined as 100%, and the relative enzyme activities at various temperatures were calculated accordingly. To evaluate temperature stability, enzyme solutions were incubated at 40 °C, 45 °C, and 50 °C for different durations in phosphate buffer (100 mM, pH 7.0) without a substrate, followed by introduction into the initial reaction system for catalytic reactions to determine the residual activity and temperature stability. The treated enzymes were then introduced into the reaction system to determine the residual activity. The activity of the unheated enzyme was considered to be 100% when calculating the relative enzyme activity.

#### 2.8.2. Determination of Optimum pH and pH Stability

At the optimum temperature, citric acid–sodium hydroxide buffer (pH 4.0–7.0) and glycine–sodium hydroxide buffer (pH 8.0–11.0) were used to adjust the pH of the reaction system. The highest enzyme activity measured was defined as 100%, and the relative enzyme activities at different pH levels were calculated. To assess pH stability, the CCD1 enzyme was incubated in 100 mM citric acid–sodium hydroxide buffer (pH 4.0–7.0) and glycine–sodium hydroxide buffer (pH 8.0–11.0) at 4 °C for 8 h. Subsequently, the treated enzyme was introduced into the reaction system to determine the residual activity. The activity of the untreated enzyme was defined as 100% for calculating the relative enzyme activity.

#### 2.8.3. Influence of Ethanol Concentration and Fe^2+^ Concentration on CCD1 Enzymatic Activity

Ethanol (0–25% *v*/*v*) and Fe^2+^ (0–1 mM) were added to the reaction system. The enzyme activity in the absence of these additives was used as a control (100% activity), and the relative enzyme activities under various conditions were calculated.

#### 2.8.4. Effects of Metal Ions and EDTA on Enzymatic Activity

The reaction system was supplemented with 1 mM of various metal ions or 5 mM EDTA to determine their effects on enzymatic activity. The enzyme activity in the absence of metal ions and EDTA was used as a control (100% activity), and the relative enzyme activities were calculated accordingly.

#### 2.8.5. Determination of Kinetic Constants

Varying concentrations of substrates were added to the reaction system, and enzyme activity was measured under optimal reaction conditions. The Michaelis–Menten constant (K_m_) and maximum velocity (V_max_) were calculated using nonlinear regression fitting in GraphPad Prism 9.0 software.

#### 2.8.6. Data Processing and Replicates

All enzyme activity assays and metal ion effect experiments were performed in three independent replicates (*n* = 3) under identical conditions. The values presented in the figures and tables represent the arithmetic mean of these measurements. Although standard deviations are not shown in some figures for clarity, the variability among replicates was generally within 10% of the mean.

### 2.9. Culture Conditions of E. coli

Luria–Bertani (LB) medium (1% tryptone, 0.5% yeast extract, 1% NaCl) was used for the molecular cloning and preparation of *E. coli* seed cultures. For β-carotene and β-ionone production, the seed culture was inoculated into 2YT medium (1.6% tryptone, 1% yeast extract, 0.5% NaCl) supplemented with 5 g/L glycerol as the initial carbon source. Cultures were grown in 250 mL shake flasks at 37 °C and 200 rpm. When the optical density at 600 nm (OD_600_) reached 0.6–0.8, IPTG was added to induce protein expression. Simultaneously, 10% (*v*/*v*) dodecane was added to the culture to capture volatile products (β-ionone). The cultures were then incubated at 30 °C and 200 rpm for an additional 48 h. Luria–Bertani (LB) medium (1% tryptone, 0.5% yeast extract, 1% NaCl) was utilized for molecular cloning and the preparation of *E. coli* seed solutions. To produce β-carotene and β-ionone, the seed culture was inoculated into 2YT medium (1.6% tryptone, 1% yeast extract, 0.5% NaCl) with 5 g/L of glycerol as the initial carbon source. This culture was maintained in a 250 mL shake flask at 37 °C and 200 rpm. Upon reaching an optical density at 600 nm (OD_600_) of 0.6–0.8, appropriate concentrations of IPTG were added. Simultaneously, 10% (*v*/*v*) dodecane was introduced to collect volatile products (β-ionone). The cultures were then further incubated at 30 °C and 200 rpm for an additional 48 h.

### 2.10. Detection of β-Ionone Concentration Using HPLC

β-Ionone concentrations were quantitatively analyzed using an HPLC 1200 system equipped with a C18 column. The mobile phase consisted of distilled water (A) and methanol (B) in a ratio of 15:85. Chromatographic separation was performed for 15 min at a flow rate of 1 mL/min, with the column temperature maintained at 30 °C. Detection was achieved by monitoring absorbance at 300 nm. The optimal temperature, pH, stability, and effects of Fe^2+^ and ethanol on enzyme activity are expressed as percentages of residual enzymatic activity relative to control conditions.

## 3. Results and Discussion

### 3.1. Gene Mining and Screening of CCD1

Carotenoid cleavage dioxygenase (CCD) enzymes are currently classified into seven families based on their functions and characteristics: CCD1, CCD2, CCD4, CCD7, CCD8, CCD10, and NCEDs. Research has shown that the CCD1 enzyme family demonstrates superior specificity for carotenoid catalytic cleavage compared to other CCD enzyme families, which directed our focus towards CCD1 enzymes. Based on the reported highly catalytic PhCCD1 sequence for carotenoids [27], potential CCD1 genes were identified using BLAST analysis on the NCBI database (BLAST+ version 2.16.0). Two CCD1 genes were selected and synthesized: *OeCCD1* (XM_023040081.1) from olive (*Olea europaea*) and *InCCD1* (XM_019295309.1) from morning glory (*Ipomoea nil*).

**Figure 1:**
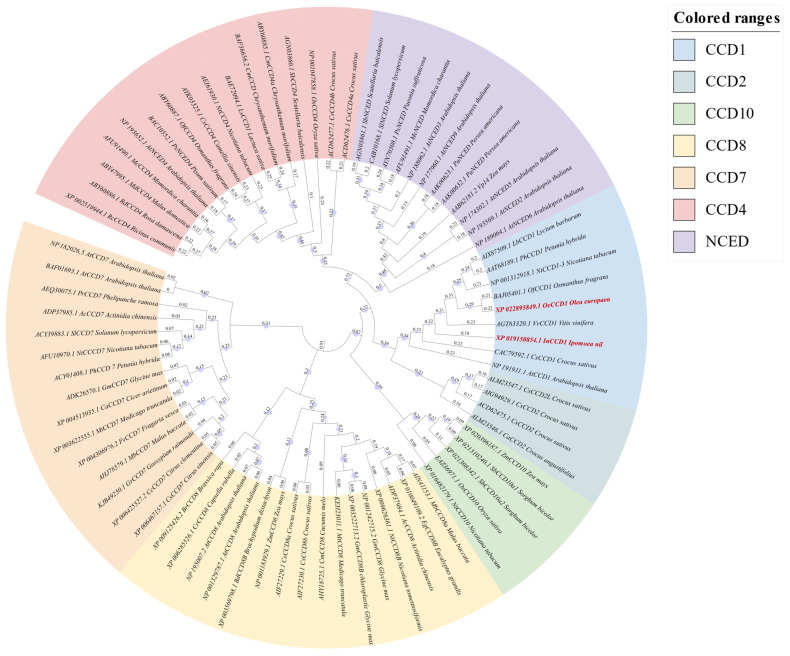
Evolutionary tree of deduced amino acid sequences of CCDs from *Olea europaea*, *Ipomoea nil*, and other plant species. The tree was generated using the MEGA 11 program and neighbor-joining algorithm. The resulting tree was bootstrap analyzed with 1000 replicates. The red bold indicates the *OeCCD1* gene and *InCCD1* gene identified in this study. The GenBank accession numbers for the sequences are shown on the branches.

A phylogenetic tree was constructed using MEGA 11.0 software based on the neighbor-joining method, incorporating *OeCCD1*, *InCCD1*, and other CCD enzymes from various sources (Figure 1). The analysis confirmed the classification of CCD enzymes into seven families: CCD1, CCD2, CCD4, CCD7, CCD8, CCD10, and NCEDs. The phylogenetic analysis revealed close evolutionary relationships between the selected CCD1 enzymes and previously characterized CCD1 enzymes from other plant species. Specifically, *OeCCD1* was closely related to *OfCCD1* from *Osmanthus grans*, and *InCCD1* was closely related to *CsCCD1* from *Crocus sativus*.

Sequence analysis revealed that the *OeCCD1* gene is 1629 bp long, encoding 542 amino acids. The *InCCD1* gene is 1704 bp long, encoding 567 amino acids. A multiple sequence alignment was performed comparing these two CCD1 sequences with the well-characterized *PhCCD1* from *Petunia hybrids*, which is known for its high catalytic efficiency towards carotenoid substrates (Figure S1). The analysis demonstrated a high degree of similarity among CCD1 sequences from different plant sources. Specifically, *OeCCD1* showed 79.62% similarity and *InCCD1* showed 75.39% similarity.

### 3.2. Optimization of CCD1 Expression Conditions

Previous studies have reported challenges in achieving a soluble expression of plant-derived CCD proteins [27]. To enhance the soluble expression of the synthesized CCD1 proteins, we employed two strategies: (1) the addition of a GST tag to the original gene sequence by assembling both CCD1 genes in the plasmid pGEX-2t-1, a vector containing a t7 promoter, and a GST tag; and (2) the optimization of the expression conditions. Furthermore, considering that CCD1 is a non-heme iron-dependent enzyme [30] and that its active center may depend on Fe^2+^, we investigated the effect of Fe^2+^ on enzyme activity. These optimizations were carried out in LB medium.

#### 3.2.1. Optimization of OeCCD1 Expression Conditions

**Figure 2:**
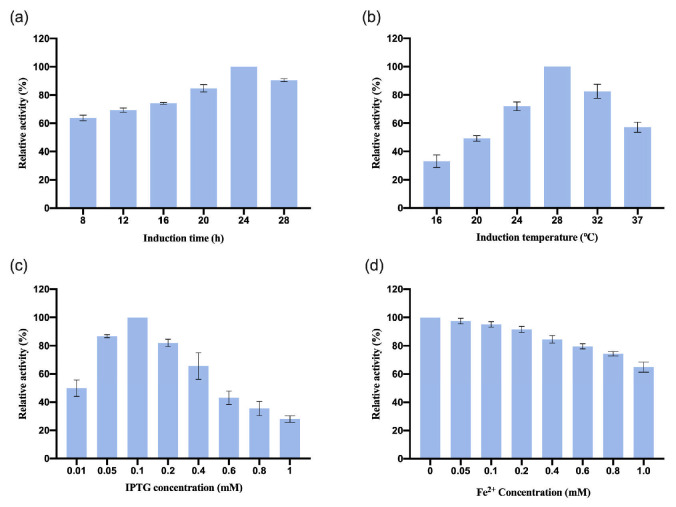
Optimization of *OeCCD1* expression conditions. (**a**) Effect of induction time on the expression of *OeCCD1*. (**b**) Effect of temperature on the expression of *OeCCD1*. (**c**) Effect of IPTG concentration on the expression of *OeCCD1*. (**d**) Effect of Fe^2+^ concentration on the expression of *OeCCD1*. The highest activity was defined as 100%. These activities are expressed as relative values.

After we assembled *OeCCD1* in the plasmid pGEX-2t-1, the recombinant plasmid pGEX-2T-*OeCCD1* was transformed into *E. coli* BL21 strains and the induction conditions were optimized. The results are shown in Figure 2. The results show that the optimal induction time for *OeCCD1* was 24 h. When the induction temperature was below 24 °C, the relative enzyme activity was less than 60%. The relative enzyme activity was highest when the induction temperature reached 28 °C. The expression of *OeCCD1* depended on IPTG, with the highest enzyme activity achieved at 0.1 mM IPTG. The enzyme activity of *OeCCD1* decreased with increasing IPTG concentration, and excessive IPTG significantly reduced the enzyme activity of *OeCCD1*. In addition, the concentration of Fe^2+^ had a significant inhibitory effect on the enzyme activity of *OeCCD1*; at 1 mM Fe^2+^ concentration, the enzyme activity of *OeCCD1* was less than 60% of the maximum enzyme activity. While CCD1 has previously been characterized as Fe^2+^-dependent [30], our results reveal a significant reduction in *OeCCD1* activity at 1 mM Fe^2+^ concentration (Figure 2d). This finding suggests that *OeCCD1* does not depend on exogenous Fe^2+^ and that endogenous trace-metal ions present in the culture medium may be sufficient for its enzymatic function. The discrepancy with earlier findings could stem from differences in enzyme conformation, expression systems, or metal cofactor incorporation efficiencies.

#### 3.2.2. Optimization of InCCD1 Expression Conditions

**Figure 3:**
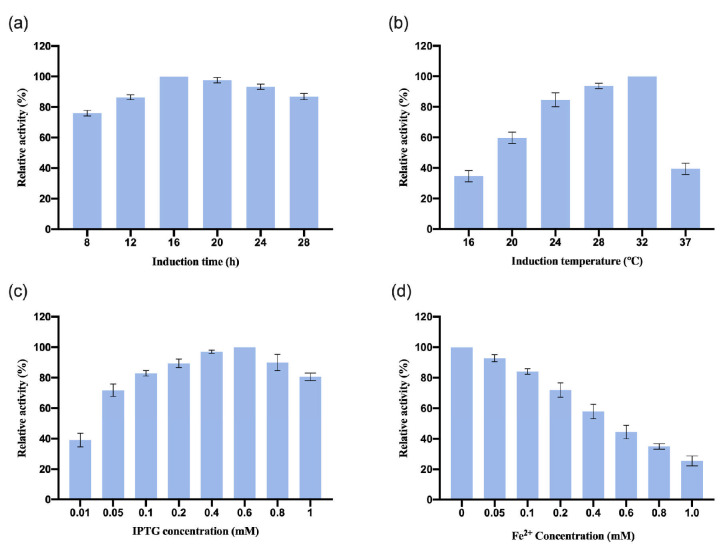
Optimization of *InCCD1* expression conditions. (**a**) Effect of induction time on the expression of *InCCD1*. (**b**) Effect of temperature on the expression of *InCCD1*. (**c**) Effect of IPTG concentration on the expression of *InCCD1*. (**d**) Effect of Fe^2+^ concentration on the expression of *InCCD1*. The highest activity was defined as 100%. These activities are expressed as relative values.

The recombinant plasmid pGEX-2T-*InCCD1* was transformed into the *E. coli* BL21 strain, and the induction conditions were also optimized. The results are shown in Figure 3. The results indicate that the optimal induction time for *InCCD1* was 16 h. The relative enzyme activity was highest when the induction temperature reached 32 °C. The expression of *InCCD1* depended on IPTG, with the maximum enzyme activity achieved at 0.6 mM IPTG. The enzyme activity of *InCCD1* decreased with increasing concentrations of IPTG, and excessive IPTG significantly reduced the enzyme activity of *InCCD1*. Additionally, the concentration of Fe^2+^ had a notable inhibitory effect on *InCCD1* enzyme activity; at a Fe^2+^ concentration of 1 mM, the enzyme activity of *InCCD1* was less than 40% of the maximum activity. Similar to *OeCCD1*, the enzymatic activity of *InCCD1* was notably suppressed at 1 mM Fe^2+^ concentration (Figure 3d). This observation implies that minimal trace metal availability in *E. coli* expression systems may suffice for catalytic function without additional supplementation.

### 3.3. Purification of CCD1 Protein

**Figure 4:**
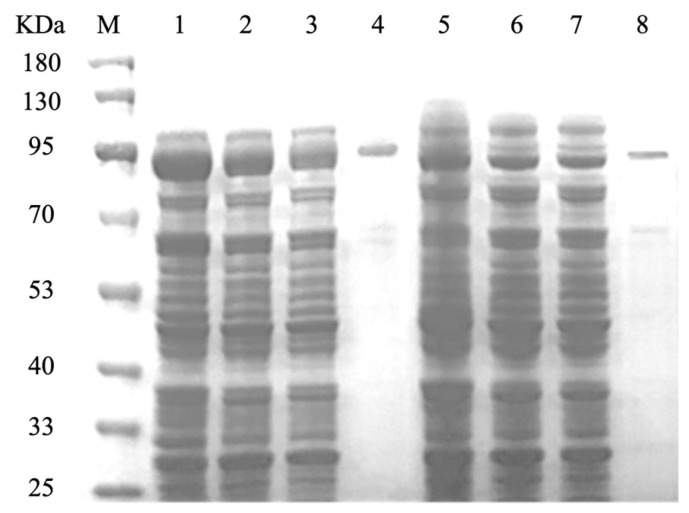
SDS-PAGE of the recombinant CCD1. M: protein marker; lane 1: whole cell lysate of the recombinant *OeCCD1*; lane 2: the crude extract of the recombinant *OeCCD1*; lane 3: the precipitation of the recombinant *OeCCD1*; lane 4: purified *OeCCD1*; lane 5: whole cell lysate of the recombinant *InCCD1*; lane 6: the crude extract of the recombinant *InCCD1*; lane 7: the precipitation of the recombinant *InCCD1*; lane 8: purified *InCCD1*.

The recombinant strains OeCCD1 and InCCD1 were cultured under their respective optimal expression conditions, and the crude enzyme solutions were collected. The crude enzyme solutions were purified using a GST-tagged affinity chromatography column. The collected elution was analyzed for purification results via SDS-PAGE, as shown in Figure 4. The molecular weights of the protein single bands for OeCCD1 and InCCD1 were approximately 87.43 kDa and 89.55 kDa, respectively, which are consistent with the predicted molecular weights.

### 3.4. Study of the Enzymatic Properties of CCD1

#### 3.4.1. Study of the Enzymatic Properties of OeCCD1

**Figure 5:**
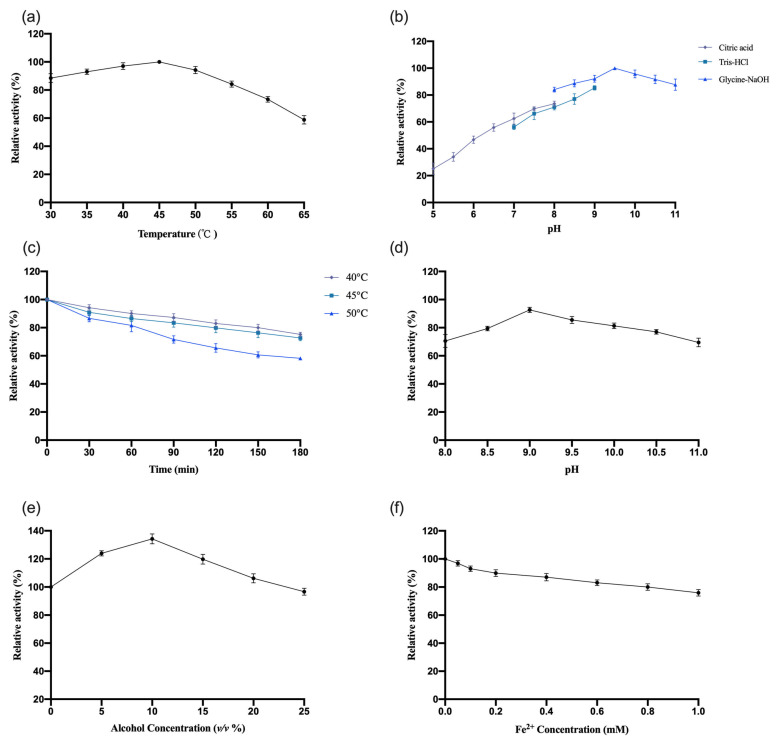
The effects of pH and temperature on the activity and stability of the recombinant *OeCCD1*, as well as the effects of alcohol and Fe^2+^ concentration on the enzymatic activity of *OeCCD1*. (**a**) Effect of pH on the enzymatic activity of *OeCCD1*. (**b**) Effect of temperature on the enzymatic activity of *OeCCD1*. (**c**) The pH stability of the enzyme *OeCCD1*. (**d**) The thermostability of the enzyme *OeCCD1*; the residual activity was monitored, while the enzyme was incubated at 40, 45, and 50 °C. The initial activity was defined as 100%. (**e**) Effect of alcohol concentration on the enzymatic activity of *OeCCD1*. (**f**) Effect of Fe^2+^ concentration on the enzymatic activity of *OeCCD1*. These activities are expressed as relative values.

The enzymatic properties of recombinant *OeCCD1* were characterized using the purified enzyme, with β-apo-8′-carotenal as the substrate. The results are shown in Figure 5. This study indicates that the optimal reaction temperature for the *OeCCD1* enzyme was 45 °C, maintaining over 80% of maximal activity within the temperature range of 30–55 °C. The enzyme activity of *OeCCD1* was measured in buffer solutions with a pH ranging from 5.0 to 11.0 (pH 5.0–8.0: 0.5 M citrate-Na_2_HPO_4_ buffer; pH 7.0–9.0: 0.5 M Tris-HCl buffer; pH 8.0–11.0: 0.5 M Gly-NaOH buffer). Recombinant *OeCCD1* exhibited higher activity in Gly-NaOH buffer, with an optimal pH of 9.5. Within the pH range of 8.0–11.0, its activity remained above 80% of the maximum enzyme activity. Acidic conditions were unfavorable for the expression of recombinant *OeCCD1*, as its activity dropped below 40% of the maximum enzyme activity when the pH was below 6.0. After purification, *OeCCD1* retained more than 60% of its maximum enzyme activity after being incubated at 40 °C, 45 °C, and 50 °C for 3 h without a substrate. The recombinant *OeCCD1* demonstrated good pH stability; after being incubated at 4 °C for 8 h within the pH range of 8.0–11.0, its residual activity remained above 70% of the initial enzyme activity. Additionally, the recombinant *OeCCD1* showed good tolerance to ethanol; the enzyme activity reached its peak with 10% ethanol in the reaction system, and when the ethanol concentration reached 25%, the enzyme activity was similar to that of the enzyme activity without ethanol. The addition of Fe^2+^ in the reaction system had a certain inhibitory effect on *OeCCD1* enzyme activity, with the enzyme activity measuring around 80% of that without Fe^2+^ when 1 mM Fe^2+^ was added. This phenomenon may be due to the trace elements present in the growth medium being sufficient for CCD1 expression, which aligns with the reported enzymatic properties of *HaCCD1* [31] and *MnCCD1* [32].

#### 3.4.2. Study of the Enzymatic Properties of InCCD1

**Figure 6:**
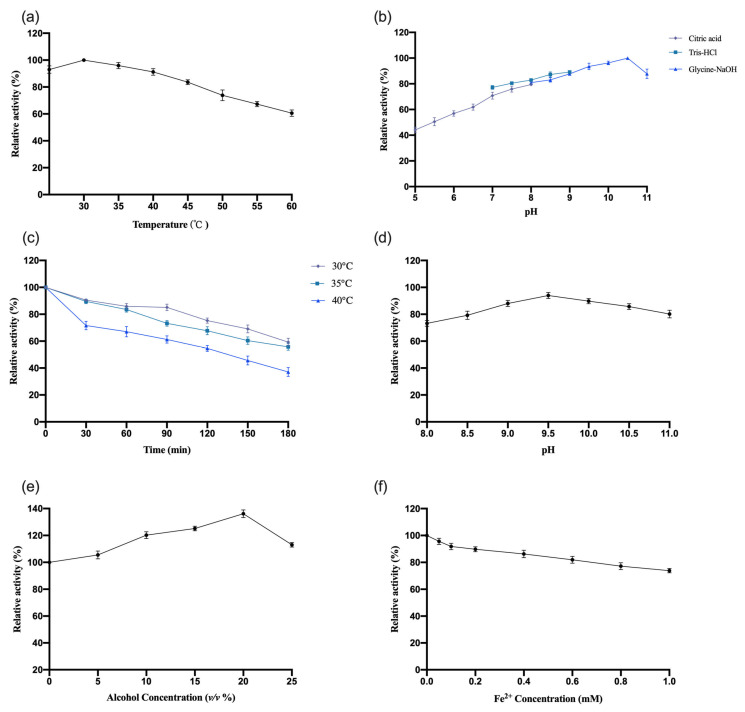
The effects of pH and temperature on the activity and stability of the recombinant *InCCD1*, as well as the effects of alcohol and Fe^2+^ concentration on the enzymatic activity of *InCCD1*. (**a**) Effect of pH on the enzymatic activity of *InCCD1*. (**b**) Effect of temperature on the enzymatic activity of *InCCD1*. (**c**) The pH stability of the enzyme *InCCD1*. (**d**) The thermostability of the enzyme *InCCD1*; the residual activity was monitored, while the enzyme was incubated at 30, 35, and 40 °C. The initial activity was defined as 100%. (**e**) Effect of alcohol concentration on the enzymatic activity of *InCCD1*. (**f**) Effect of Fe^2+^ concentration on the enzymatic activity of *InCCD1*. These activities are expressed as relative values.

The enzymatic properties of recombinant *InCCD1* were investigated using the same methodology as previously described for *OeCCD1*. The results are shown in Figure 6. The recombinant *InCCD1* exhibited higher activity in the Gly-NaOH buffer, with an optimal pH of 10.5. Within the pH range of 8.0–11.0, the activity maintained over 80% of the maximum enzyme activity. After purification, the *InCCD1* enzyme displayed poor thermal stability under conditions without a substrate; after incubation at 30 °C, 35 °C, and 40 °C for 3 h, the remaining activity was less than 80% of the maximum enzyme activity at 30 °C and only about 40% of the maximum enzyme activity after 3 h at 40 °C. The recombinant *InCCD1* enzyme also exhibited good pH stability; following an 8 h incubation at 4 °C within the pH range of 8.0–11.0, the activity consistently remained above 70% of the initial enzyme activity. The recombinant *InCCD1* enzyme also showed good tolerance to ethanol, achieving peak activity at 20% ethanol concentration. When the ethanol concentration reached 25%, the activity of *InCCD1* was comparable to that without ethanol. The addition of Fe^2+^ in the reaction system had a certain inhibitory effect on the activity of *InCCD1*, with enzyme activity at approximately 80% of the activity when no Fe^2+^ was added at a concentration of 1 mM.

#### 3.4.3. Effects of Common Metal Ions and EDTA on the Enzymatic Activities of Recombinant CCD1 Enzymes

The impact of adding metal ions and EDTA at a final concentration of 1 mM on the enzyme activities of *OeCCD1* and *InCCD1* was measured, and the results are shown in Table 1. In total, 1 mM of Fe^3+^, Ca^2+^, Ba^2+^, Sr^2+^, K^+^, Ni^2+^, Co^2+^, Al^3+^, Li^+^, Mg^2+^, NH^4+^, and EDTA all slightly promoted the activity of *OeCCD1*. However, 1 mM Fe^2+^ inhibited the activity of *OeCCD1*, with the remaining enzyme activity being less than 80% of the initial activity. 1 mM Co^2+^ and Zn^2+^ slightly promoted the activity of *InCCD1*, while other metal ions inhibited its activity. Among them, 1 mM Fe^2+^, Ba^2+^, and Mg^2+^ strongly inhibited the activity of *InCCD1*, leaving less than 80% of its initial activity. Additionally, 1 mM EDTA reduced the activity of *InCCD1* by about 20% compared to its initial activity.

#### 3.4.4. Determination of CCD1 Kinetic Constants

The kinetic constants of *OeCCD1* and *InCCD1* were determined using β-apo-8′-carotenal as the substrate. Kinetic parameters were determined via nonlinear fitting of the Michaelis–Menten equation using GraphPad Prism 9.0. As shown in Table 2, the K_m_ for *OeCCD1* was 0.82 mM and the V_max_ was 2.30 U/mg, with a catalytic efficiency (k_cat_/K_m_) of 4.09 mM^−1^·s^−1^; the K_m_ for *InCCD1* was 0.69 mM, the V_max_ was 1.22 U/mg, and the k_cat_/K_m_ was 2.64 mM^−1^·s^−1^.

### 3.5. Substrate Specificity of CCD1 In Vivo

Due to the poor water solubility of most carotenoids, preparing micellar solutions are challenging. To overcome this limitation, CCD1 plasmids were expressed in *E. coli* chassis cells engineered to accumulate zeaxanthin and β-carotene. These cells were cultured under optimal expression conditions to assess CCD1 enzyme activity on these three carotenoids in vivo.

The plasmids pETDuet-*OeCCD1* and pETDuet-*InCCD1* were transformed into *E. coli* cells accumulating β-carotene and zeaxanthin. *E. coli* cells transformed with empty plasmids served as controls. Carotenoid retention times were verified against standard substances.

This study reveals that cultures of *E. coli* chassis cells carrying CCD1 plasmids exhibit noticeably lighter coloration compared to those with empty plasmids for β-carotene and zeaxanthin accumulators. Based on these observations, we hypothesized that *OeCCD1* and *InCCD1* demonstrate cleavage activity towards β-carotene and zeaxanthin in vivo. To test this hypothesis, a HPLC analysis was employed to detect carotenoid degradation in the fermentation broth.

The experimental results presented in Figures S2 and S3 show that, compared to cultures of β-carotene and zeaxanthin accumulating chassis cells with empty plasmids, those carrying CCD1 plasmids exhibited significantly reduced carotenoid peak areas.

**Figure 7:**
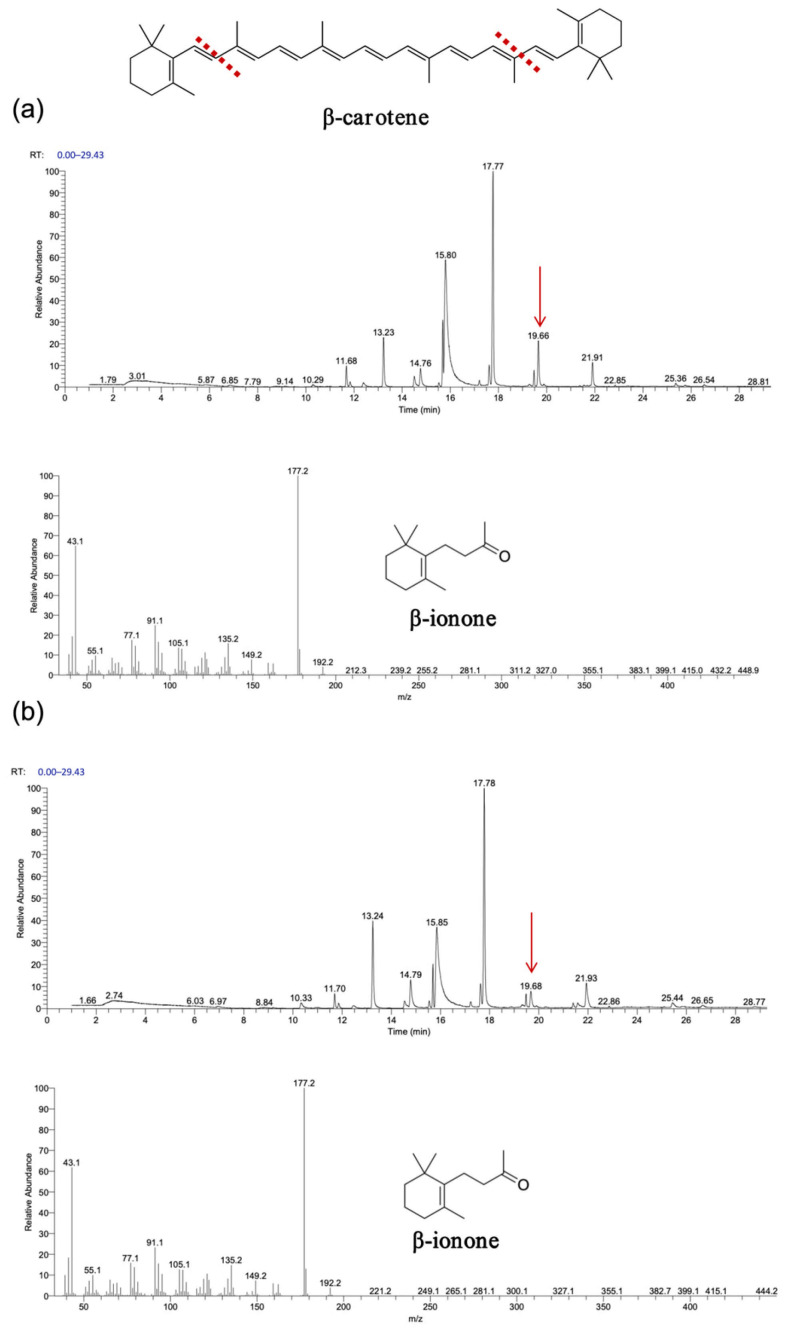
Functional expression of *OeCCD1* and *InCCD1* in β-carotene-producing *E. coli* cells. (**a**) GC–MS analysis of the cleavage products of β-carotene synthesized using *OeCCD1*. (**b**) GC–MS analysis of the cleavage products of β-carotene synthesized using *InCCD1*. The mass spectra of peak was identified as β-ionone with the standard. The red arrow indicates the position of the cleavage product peak, and the red line represents the putative cleavage site of carotenoids catalyzed by CCD1.

**Figure 8:**
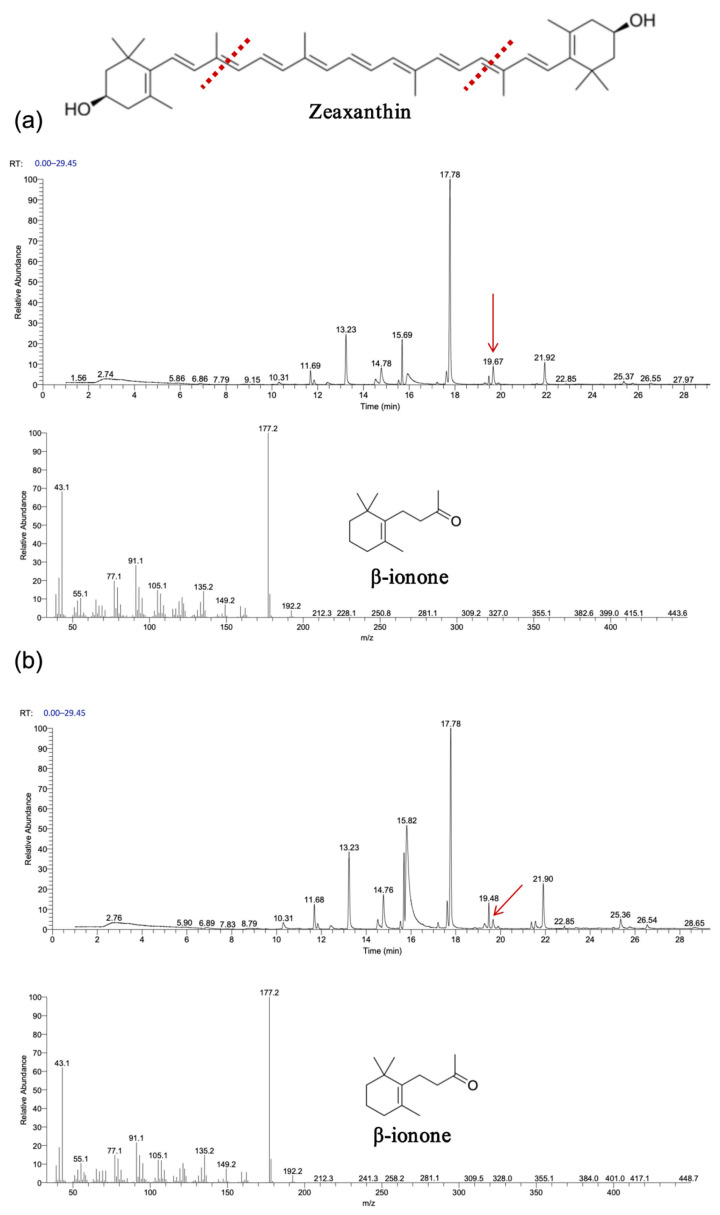
Functional expression of *OeCCD1* and *InCCD1* in Zeaxanthin-producing *E. coli* cells. (**a**) GC–MS analysis of the cleavage products of Zeaxanthin synthesized using *OeCCD1*. (**b**) GC–MS analysis of the cleavage products of Zeaxanthin synthesized using *InCCD1*. The mass spectra of peak was identified as β-ionone with the standard. The red arrow indicates the position of the cleavage product peak, and the red line represents the putative cleavage site of carotenoids catalyzed by CCD1.

As shown in Figure 7 and Figure 8, gas chromatography–mass spectrometry (GC–MS) analysis identified β-ionone as a volatile compound produced in the fermentation broth of chassis cells harboring the CCD1-expressing plasmid. This result is consistent with previous findings [28,31,32]. Interestingly, CCD1 is theoretically capable of cleaving zeaxanthin to generate 3-hydroxy-β-ionone. However, considering that β-carotene is the biosynthetic precursor of zeaxanthin, it is likely that zeaxanthin was not substantially utilized in vivo. Instead, both CCD1 enzymes may preferentially target β-carotene, leading to reduced zeaxanthin accumulation and the predominant production of β-ionone.

### 3.6. In Vitro Substrate Specificity Study of CCD1

To comprehensively assess CCD1’s substrate specificity, six carotenoids were selected as substrates: β-carotene, zeaxanthin, lycopene, lutein, astaxanthin, and trans-retinal. Following enzymatic reactions, a GC-MS analysis was performed to investigate the cleavage products. The resulting data were compared with standard spectra.

**Figure 9:**
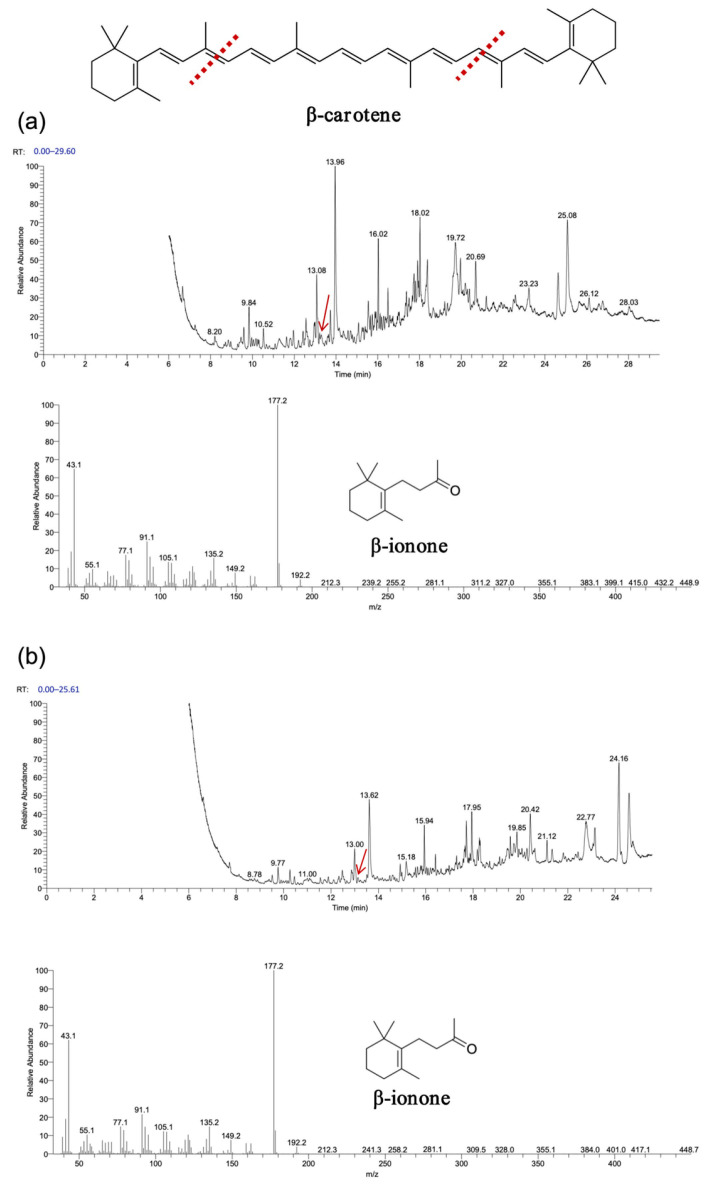
β-carotene was oxidatively cleaved using *OeCCD1* and *InCCD1* to yield volatile products in vitro. (**a**) β-carotene was oxidatively cleaved using *OeCCD1* to yield volatile products in vitro. (**b**) β-carotene was oxidatively cleaved using *InCCD1* to yield volatile products in vitro. The red arrow indicates the position of the cleavage product peak, and the red line represents the putative cleavage site of carotenoids catalyzed by CCD1.

**Figure 10:**
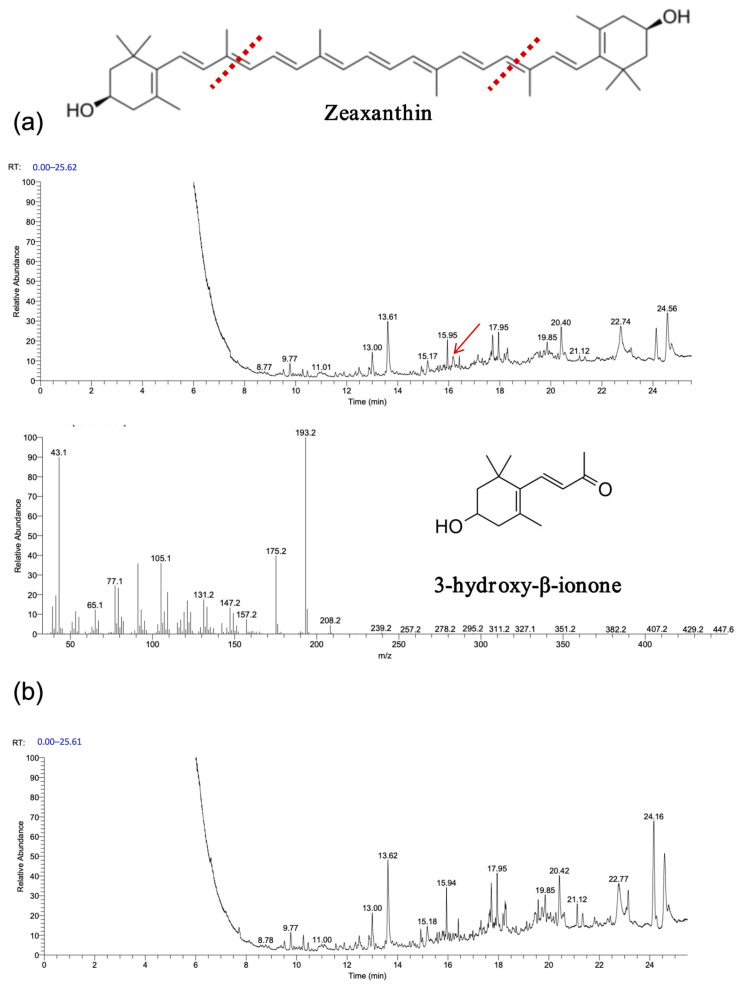
Zeaxanthin was only oxidatively cleaved using *OeCCD1* to yield volatile products in vitro. (**a**) Zeaxanthin was oxidatively cleaved using *OeCCD1* to yield volatile products in vitro. (**b**) Zeaxanthin was not oxidatively cleaved using *InCCD1* to yield volatile products in vitro. The red arrow indicates the position of the cleavage product peak, and the red line represents the putative cleavage site of carotenoids catalyzed by CCD1.

**Figure 11:**
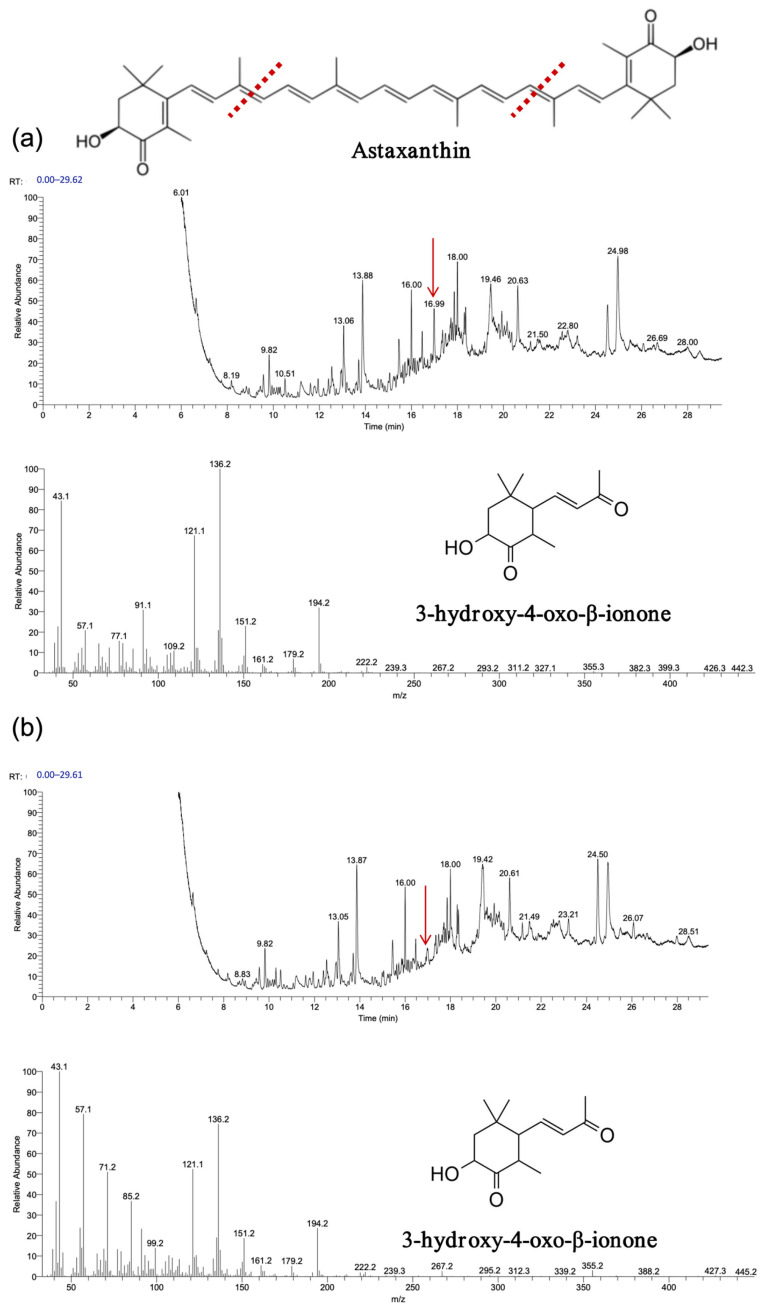
Astaxanthin was oxidatively cleaved using *OeCCD1* and *InCCD1* to yield volatile products in vitro. (**a**) Astaxanthin was oxidatively cleaved using *OeCCD1* to yield volatile products in vitro. (**b**) Astaxanthin was oxidatively cleaved using *InCCD1* to yield volatile products in vitro. The red arrow indicates the position of the cleavage product peak, and the red line represents the putative cleavage site of carotenoids catalyzed by CCD1.

The experimental results, depicted in Figure 9, Figure 10 and Figure 11, demonstrate that recombinant *OeCCD1* and *InCCD1* enzymes catalyzed the cleavage of β-carotene and astaxanthin at the 9,10 (9′,10′) positions, producing β-ionone and 3-hydroxy-4-oxo-β-ionone, respectively. However, no catalytic activity was observed on the 9,10 (9′,10′) positions of lutein or trans-retinal.

Interestingly, recombinant *OeCCD1* catalyzed the cleavage of zeaxanthin at the 9,10 (9′,10′) positions to generate 3-hydroxy-β-ionone, while recombinant *InCCD1* enzyme did not produce β-ionone or related substances from zeaxanthin. This observation indicates that the recombinant *InCCD1* enzyme is unable to oxidatively cleave zeaxanthin in vitro, which further supports the hypothesis proposed in the previous section.

### 3.7. Evaluation of CCD1 Enzymes for β-Ionone Biosynthesis in Engineered E. coli Under IPTG Induction

**Figure 12:**
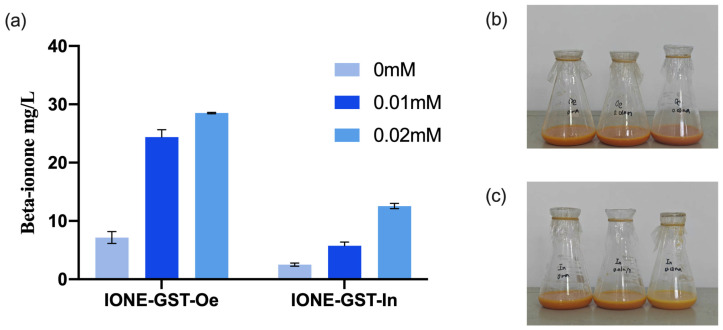
β-Ionone production and growth performance of engineered de novo β-ionone synthesis strains under varying IPTG induction conditions. (**a**) Effect of IPTG concentration on β-ionone yield (**b**) The growth status of the strain IONE-GST-Oe under different IPTG concentrations (from left to right: 0 mM, 0.01 mM, 0.02 mM). (**c**) The growth status of the strain IONE-GST-In under different IPTG concentrations (from left to right: 0 mM, 0.01 mM, 0.02 mM).

To evaluate the potential applications of CCD1 variants, two types of CCD1 were introduced into the high-yielding β-carotene *E. coli* chassis cell MVABETA12 to construct two de novo synthetic β-ionone strains (IONE-GST-Oe, IONE-GST-In) [33]. Given that previous studies have shown that IPTG concentration significantly influences the production of β-ionone in engineered strains [28], we investigated three different IPTG concentrations to optimize β-ionone production in these strains. As shown in Figure 12, IONE-GST-In achieved a β-ionone yield of 12.84 mg/L at an IPTG concentration of 0.02 mM, while IONE-GST-Oe demonstrated superior performance with a yield of 28.37 mg/L under the same IPTG concentration. These results collectively demonstrate that these two CCD1 variants possess application potential for β-ionone production.

## 4. Conclusions

In this study, we identify and functionally characterize two CCD1 homologs—*OeCCD1* from the woody plant *Olea europaea*, and *InCCD1* from the herbaceous plant *Ipomoea nil*—to explore functional diversity in carotenoid cleavage enzymes. Both enzymes catalyzed the oxidative cleavage of β-carotene at the 9,10 (or 9′,10′) double-bond to produce β-ionone. However, only *OeCCD1* exhibited in vitro activity on zeaxanthin, while *InCCD1* showed no detectable cleavage of this substrate.

Kinetic analysis using β-apo-8′-carotenal as a substrate revealed that *OeCCD1* had a K_m_ of 0.82 mM, a V_max_ of 2.30 U/mg, and a catalytic efficiency (k_cat_/K_m_) of 4.09 mM^−1^·s^−1^, outperforming *InCCD1* (K_m_ = 0.69 mM; V_max_ = 1.22 U/mg; k_cat_/K_m_ = 2.64 mM^−1^·s^−1^). Under optimized conditions, engineered β-carotene *E. coli* cell expressing *OeCCD1* achieved a β-ionone titer of 28.37 mg/L, more than double that of the *InCCD1* strain (12.84 mg/L). Furthermore, *OeCCD1* exhibited greater thermal and pH stability, retaining over 80% activity between 30–55 °C and pH 8.0–11.0. These results not only highlight the superior catalytic performance and robustness of *OeCCD1*, but also suggest that plant lineage (woody vs. herbaceous) may influence CCD1 enzyme properties. This study expands the known repertoire of CCD1 enzymes and highlights *OeCCD1* as a promising candidate for constructing microbial cell factories for high-yield β-ionone production.

These findings provide a foundation for the development of efficient microbial cell factories for the production of natural apocarotenoids. Future research should focus on further engineering these enzymes to enhance the production of β-ionone.

## Figures and Tables

**Table 1:** Effects of cations and reagents (1 mM) on the activity of purified CCD1.

Cation of Reagent	*OECCD1* Relative Activity (%)	*INCCD1* Relative Activity (%)
Control	100.00	100.00
Fe^2+^	78.65	79.83
Fe^3+^	103.92	92.93
Ca^2+^	102.39	87.16
Ba^2+^	101.31	73.01
Sr^2+^	101.02	90.44
K^+^	100.82	89.48
Cu^2+^	85.57	96.50
Ni^2+^	101.51	95.62
Co^2+^	101.48	103.94
Zn^2+^	98.64	106.59
Al^3+^	102.67	99.10
Na^+^	98.88	96.14
Li^+^	100.83	95.00
Mg^2+^	100.57	78.23
NH^4+^	100.18	97.06
Mn^2+^	94.86	99.35
EDTA	101.61	80.93

**Table 2:** Kinetic constant of CCD1 when β-apo-8′-carotenal is used as the substrate.

Enzyme	K_m_ (mM)	V_max_ (U/mg)	k_cat_ (s^−1^)	k_cat_/K_m_ (mM^−1^·s^−1^)
*OeCCD1*	0.82	2.30	3.35	4.09
*InCCD1*	0.69	1.22	1.82	2.64

## Data Availability

Data are contained within the article.

## Supplementary Materials

The following supporting information can be downloaded at: https://www.mdpi.com/article/10.3390/biology14070752/s1. Strains and plasmids used in this study (Table S1); The amino acid sequence alignment between carotenoid cleavage dioxygenase *PhCCD1*, *OeCCD1* and *InCCD1*. *PhCCD1* from *Petunia hybrida* (AAT68189.1), *OeCCD1* from *Olea europaea* (XP_022895849.1), *InCCD1* from *Ipomoea nil* (XP_019150854.1). (Figure S1); HPLC analysis of β-carotene cleaved by CCD1 in bacterial cultures. (Figure S2); HPLC analysis of zeaxanthin cleaved by CCD1 in bacterial cultures. (Figure S3).

## References

[1] Hou X., Rivers J., León P., McQuinn R.P., Pogson B.J. (2016). Synthesis and Function of Apocarotenoid Signals in Plants. Trends Plant Sci..

[2] Spudich J.L., Yang C.S., Jung K.H., Spudich E.N. (2000). Retinylidene proteins: Structures and functions from archaea to humans. Annu. Rev. Cell Dev. Biol..

[3] Pereira da Costa D., Campos Miranda-Filho K. (2019). The use of carotenoid pigments as food additives for aquatic organisms and their functional roles. Rev. Aquac..

[4] Walter M.H., Floss D.S., Strack D. (2010). Apocarotenoids: Hormones, mycorrhizal metabolites and aroma volatiles. Planta.

[5] Czajka J.J., Nathenson J.A., Benites V.T., Baidoo E.E.K., Cheng Q.S., Wang Y.C., Tang Y.J.J. (2018). Engineering the oleaginous yeast *Yarrowia lipolytica* to produce the aroma compound β-ionone. Microb. Cell Fact..

[6] Serra S. (2015). Recent Advances in the Synthesis of Carotenoid-Derived Flavours and Fragrances. Molecules.

[7] Lalko J., Lapczynski A., McGinty D., Bhatia S., Letizia C.S., Api A.M. (2007). Fragrance material review on β-ionone. Food Chem. Toxicol..

[8] Aloum L., Alefishat E., Adem A., Petroianu G. (2020). Ionone Is More than a Violet’s Fragrance: A Review. Molecules.

[9] Cataldo V.F., Lopez J., Carcamo M., Agosin E. (2016). Chemical vs. biotechnological synthesis of C13-apocarotenoids: Current methods, applications and perspectives. Appl. Microbiol. Biotechnol..

[10] Zhang X.S., Pei J.J., Zhao L.G., Tang F., Fang X.Y., Xie J.C. (2016). Overexpression and characterization of CCD4 from *Osmanthus fragrans* and β-ionone biosynthesis from β-carotene in vitro. J. Mol. Catal. B Enzym..

[11] Zhang C., Chen X., Lindley N.D., Too H.P. (2018). A “plug-n-play” modular metabolic system for the production of apocarotenoids. Biotechnol. Bioeng..

[12] Lopez J., Bustos D., Camilo C., Arenas N., Saa P.A., Agosin E. (2020). Engineering Saccharomyces cerevisiae for the Overproduction of beta-Ionone and Its Precursor beta-Carotene. Front. Bioeng. Biotechnol..

[13] Lu Y., Yang Q., Lin Z., Yang X. (2020). A modular pathway engineering strategy for the high-level production of beta-ionone in *Yarrowia lipolytica*. Microb. Cell Fact..

[14] Werner N., Ramirez-Sarmiento C.A., Agosin E. (2019). Protein engineering of carotenoid cleavage dioxygenases to optimize beta-ionone biosynthesis in yeast cell factories. Food Chem..

[15] Chen X., Shukal S., Zhang C. (2019). Integrating Enzyme and Metabolic Engineering Tools for Enhanced alpha-Ionone Production. J. Agric. Food Chem..

[16] Schwartz S.H., Tan B.C., Gage D.A., Zeevaart J.A., McCarty D.R. (1997). Specific oxidative cleavage of carotenoids by VP14 of maize. Science.

[17] Auldridge M.E., McCarty D.R., Klee H.J. (2006). Plant carotenoid cleavage oxygenases and their apocarotenoid products. Curr. Opin. Plant Biol..

[18] Martinez-Andujar C., Ordiz M.I., Huang Z., Nonogaki M., Beachy R.N., Nonogaki H. (2011). Induction of 9-cis-epoxycarotenoid dioxygenase in *Arabidopsis thaliana* seeds enhances seed dormancy. Proc. Natl. Acad. Sci. USA.

[19] Priya R., Siva R. (2015). Analysis of phylogenetic and functional diverge in plant nine-cis epoxycarotenoid dioxygenase gene family. J. Plant Res..

[20] Schwartz S.H., Qin X.Q., Loewen M.C. (2004). The biochemical characterization of two carotenoid cleavage enzymes from *Arabidopsis* indicates that a carotenoid-derived compound inhibits lateral branching. J. Biol. Chem..

[21] Qi Z., Tong X., Zhang Y., Jia S., Fang X., Zhao L. (2023). Carotenoid Cleavage Dioxygenase 1 and Its Application for the Production of C13-Apocarotenoids in Microbial Cell Factories: A Review. J. Agric. Food Chem..

[22] Ahrazem O., Gomez-Gomez L., Rodrigo M.J., Avalos J., Limon M.C. (2016). Carotenoid Cleavage Oxygenases from Microbes and Photosynthetic Organisms: Features and Functions. Int. J. Mol. Sci..

[23] Li F., Gong X., Liang Y., Peng L., Han X., Wen M. (2022). Characteristics of a new carotenoid cleavage dioxygenase NtCCD10 derived from Nicotiana tabacum. Planta.

[24] Zhong Y., Pan X., Wang R., Xu J., Guo J., Yang T., Zhao J., Nadeem F., Liu X., Shan H. (2020). ZmCCD10a Encodes a Distinct Type of Carotenoid Cleavage Dioxygenase and Enhances Plant Tolerance to Low Phosphate. Plant Physiol..

[25] Li T., Deng Y.J., Liu J.X., Duan A.Q., Liu H., Xiong A.S. (2021). DcCCD4 catalyzes the degradation of alpha-carotene and beta-carotene to affect carotenoid accumulation and taproot color in carrot. Plant J..

[26] Wang J., Wu B., Zhang N., Zhao M., Jing T., Wu Y., Hu Y., Yu F., Wan X., Schwab W. (2020). Dehydration-Induced Carotenoid Cleavage Dioxygenase 1 Reveals a Novel Route for beta-Ionone Formation during Tea (*Camellia sinensis*) Withering. J. Agric. Food Chem..

[27] Schwartz S.H., Qin X., Zeevaart J.A. (2001). Characterization of a novel carotenoid cleavage dioxygenase from plants. J. Biol. Chem..

[28] Ke K., Guo L., Qi Z., Dou D., Ma H., Fang X., Zhao L. (2024). Molecular cloning, expression, and biochemical characterization of carotenoid cleavage dioxygenase 1 (LbCCD1) from *Lycium barbarum*. Mol. Catal..

[29] Liu Q., Li D., Wang W., Wang D., Meng X., Wang Y. (2016). Chemical Composition and Antioxidant Activity of Essential Oils and Methanol Extracts of Different Parts from Juniperus rigida Siebold & Zucc. Chem. Biodivers..

[30] Baldermann S., Kato M., Fleischmann P., Watanabe N. (2012). Biosynthesis of alpha- and beta-ionone, prominent scent compounds, in flowers of *Osmanthus fragrans*. Acta Biochim. Pol..

[31] Qi Z., Tong X., Bu S., Pei J., Zhao L. (2022). Cloning and Characterization of a Novel Carotenoid Cleavage Dioxygenase 1 from Helianthus annuus. Chem. Biodivers..

[32] Qi Z., Fan X., Zhu C., Chang D., Pei J., Zhao L. (2022). Overexpression and Characterization of a Novel Plant Carotenoid Cleavage Dioxygenase 1 from Morus notabilis. Chem. Biodivers..

[33] Qi Z., Tong X., Ke K., Wang X., Pei J., Bu S., Zhao L. (2024). De Novo Synthesis of Dihydro-beta-ionone through Metabolic Engineering and Bacterium-Yeast Coculture. J. Agric. Food Chem..

