# Supplementary material for: Identification, Cloning, and Functional Characterization of Carotenoid Cleavage Dioxygenase (CCD) from Olea europaea and Ipomoea nil
Source: Biology (Basel). 2025 Jun 24;14(7):752. doi: 10.3390/biology14070752 (PMC12292525; doi:10.3390/biology14070752)
Supplement: Supplementary file 1 [file biology-14-00752-s001.zip › biology-3700625-supplementary.docx]

Identification, Cloning, and Functional Characterization of Carotenoid Cleavage Dioxygenase (CCD) from *Olea europaea* and *Ipomoea nil*

Kaixuan Ke ^1,2,†^, Yufeng Zhang ^1,2,†^, Xinyi Wang ^1,2^, Zhaoyan Luo ^1,2^, Yangyang Chen ^1,2^, Xianying Fang ^1,2,3,^* and Linguo Zhao ^1,3,4,^*

^1^ National Key Laboratory for the Development and Utilization of Forest Food Resources, Nanjing Forestry University, Nanjing 210037, China

^2^ College of Chemical Engineering, Nanjing Forestry University, Nanjing 210037, China

^3^ Jiangsu Co-Innovation Center of Efficient Processing and Utilization of Forest Resources, Nanjing Forestry University, Nanjing 210037, China

^4^ Jinpu Research Institute, Nanjing Forestry University, Nanjing 210037, China

* Correspondence: xianyingfang@njfu.edu.cn (X.F.); lgzhao@njfu.edu.cn (L.Z.)

^†^ These authors contributed equally to this work.

| **Table S1** Strains and plasmids used in this study. | | |
| --- | --- | --- |
| Plasmids/ Strains | Description | Source |
| *E. coli* BL21 (DE3) | F− Δ(*lac*ZYA-*arg*F)U169 *rec*A1 *end*A1 *hsd*R17 | Invitrogen |
| *E. coli* DH5α | F ^−^φ80 *lac*ZΔM15Δ(*lac*ZYA-*arg*F)U169 *end*A1 *rec*A1 *hsd*R17(r_k_ ^−^,m_k_ ^−^) *sup* E44 | Tsingke |
| *E. coli* BL21/ pEGX-2T-*OeCCD1* | *E. coli* BL21 harboring pEGX-2T-*OeCCD1* | This study |
| *E. coli* BL21/ pEGX-2T-*InCCD1* | *E. coli* BL21 harboring pEGX-2T-*InCCD1* | This study |
| *E. coli* BL21/ PAC-BETA, pETDuet-1 | *E. coli* BL21 harboring PAC-BETA and pETDuet-1 | This study |
| *E. coli* BL21/ pAC-ZEAX, pETDuet-1 | *E. coli* BL21 harboring pAC-ZEAX and pETDuet-1 | This study |
| *E. coli* BL21/ PAC-BETA，pETDuet-*OeCCD1* | *E. coli* BL21 harboring PAC-BETA，pETDuet-*OeCCD1* | This study |
| *E. coli* BL21/ PAC-BETA，pETDuet-*InCCD1* | *E. coli* BL21 harboring PAC-BETA，pETDuet-*InCCD1* | This study |
| *E. coli* BL21/ pAC-ZEAX，pETDuet-*OeCCD1* | *E. coli* BL21 harboring pAC-ZEAX，pETDuet-*OeCCD1* | This study |
| *E. coli* BL21/ pAC-ZEAX，pETDuet-*InCCD1* | *E. coli* BL21 harboring pAC-ZEAX，pETDuet-*InCCD1* | This study |
| MVABETA12 | *E. coli* DH5α harboring pMVA2302, pCD-Zu-BETA | [1] |
| IONE-GST-Oe | MVABETA12 harboring pGEX-2T-*OeCCD1* | This study |
| IONE-GST-In | MVABETA12 harboring pGEX-2T-*InCCD1* | This study |
| pGEX-2T-1 | pBR322 ori; Amp^r^ | Novagen |
| pETDuet-1 | pBR322 ori; Amp^r^ | Novagen |
| pGEX-2T-*OeCCD1* | pGEX-2T-1 harboring *OeCCD1* gene from *Olea europaea* | This study |
| pGEX-2T-*InCCD1* | pGEX-2T-1 harboring *InCCD1* gene from *Ipomoea nil* | This study |
| pETDuet-*OeCCD1* | pETDuet-1 harboring *OeCCD1* gene from *Olea europaea* | This study |
| pETDuet-*InCCD1* | pETDuet-1 harboring *InCCD1* gene from *Ipomoea nil* | This study |
| PAC-BETA | *Cm^R^*， endogenous promoter，crtE, crtB, crtI, crtY | addgene # 53272 |
| PAC-LYCipi | *Cm^R^*， endogenous promoter，crtE, ipi, crtI, crtB | addgene # 53279 |
| pAC-ZEAX | *Cm^R^*， endogenous promoter，crtE, crtB, crtI, crtY, crtZ | addgene # 53274 |
| pMVA2302 | p15A ori, CmR, PlacUV5, MTSA, T1, MBI-f, T1002, Ptrc, trGPPS | [1] |
| pCD-Zu-BETA | *Spec^R^*， endogenous promoter，crtE, crtB, crtI, crtY | [1] |

| 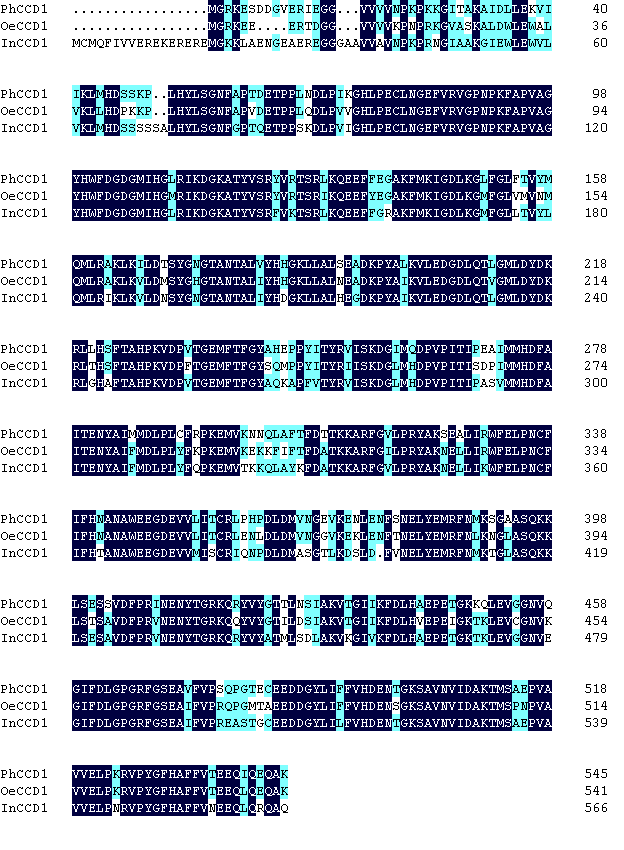 |
| --- |
| **Figure S1** The amino acid sequence alignment between carotenoid cleavage dioxygenase *PhCCD1*, *OeCCD1* and *InCCD1*. *PhCCD1* from *Petunia hybrida* (AAT68189.1), *OeCCD1* from *Olea europaea* (XP_022895849.1), *InCCD1* from *Ipomoea nil* (XP_019150854.1). |

| 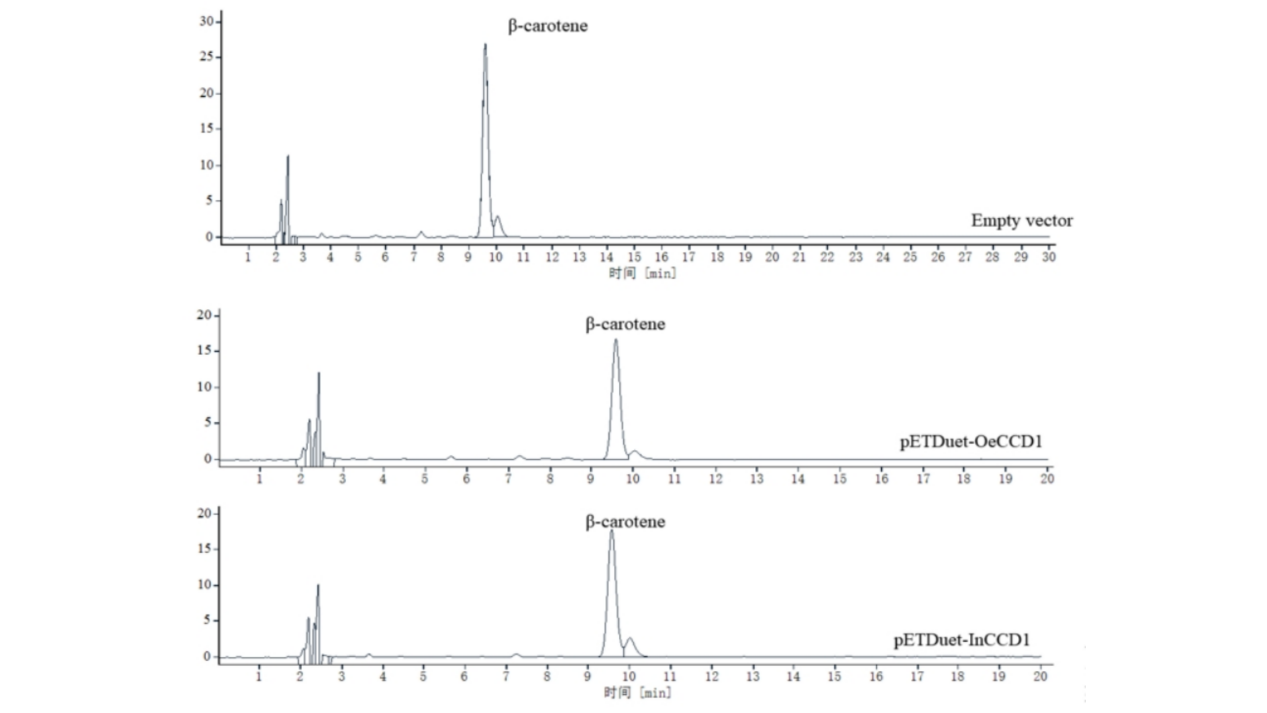 |
| --- |
| **Figure S2** HPLC analysis of β-carotene cleaved by CCD1 in bacterial cultures. |

| 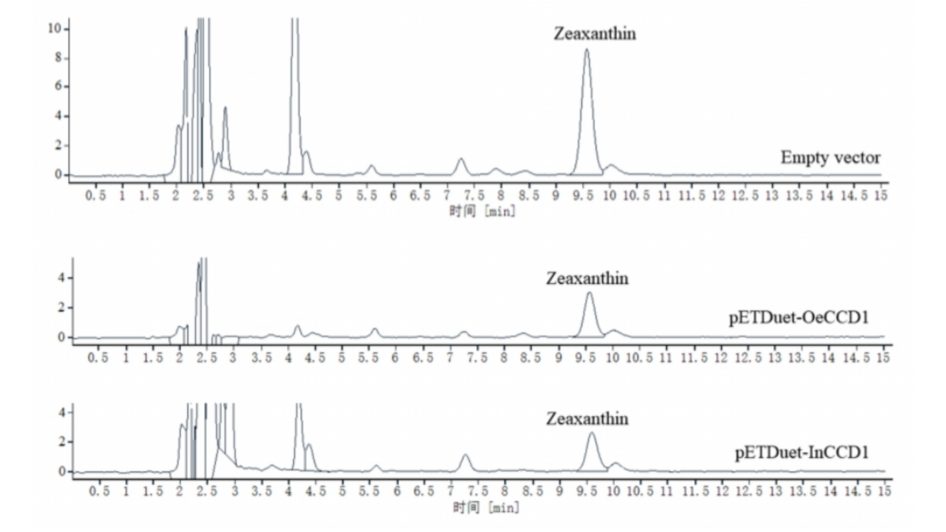 |
| --- |
| **Figure S3** HPLC analysis of zeaxanthin cleaved by CCD1 in bacterial cultures. |

**Reference：**

[1] Z. Qi, X. Tong, K. Ke, X. Wang, J. Pei, S. Bu, L. Zhao, De Novo Synthesis of Dihydro-beta-ionone through Metabolic Engineering and Bacterium-Yeast Coculture, J Agric Food Chem, 72 (2024) 3066-3076.
